# Establishment and phenotypic characterization of a rat model with a clinically relevant phenotype for chronic gouty arthritis

**DOI:** 10.3389/fimmu.2026.1752151

**Published:** 2026-04-17

**Authors:** Cunxiang Xie, Yidi Huang, Jinying Fang, Mingxuan Liu, Yingkai Gao, Luming Zhao, Hao Wang, Kaiyuan Yang, Hailong Wang

**Affiliations:** Department of Rheumatology, Dongzhimen Hospital, Beijing University of Chinese Medicine, Beijing, China

**Keywords:** bone erosion, chronic gouty arthritis, disease models, hyperuricemia, monosodium urate

## Abstract

**Background:**

The study of chronic gouty arthritis (CGA) is limited by the absence of animal models that faithfully mimic its chronic progression. Existing models fail to capture the transition from acute flares to chronic joint damage, highlighting the need for a more representative model.

**Methods:**

A novel CGA model was established in SD rats using a combined strategy. Chronic hyperuricemia was induced via a diet containing 2% potassium oxonate and 12% yeast. This was supplemented with twice-weekly gavage of hypoxanthine to simulate acute uric acid fluctuations, followed by intra-articular injections of monosodium urate (MSU) crystals into the ankle and plantar region. This protocol lasted 10 weeks. Rats were divided into control, model, and allopurinol treatment groups. Evaluations included serum uric acid monitoring, joint swelling and inflammation scores, hindlimb hanging tests, histopathology, micro-CT, and inflammatory cytokine assays.

**Results:**

The model group exhibited sustained hyperuricemia with acute fluctuations, persistent joint swelling, significantly elevated inflammation scores (P<0.0001), and reduced hanging time (P<0.0001), indicating chronic pain and functional impairment. Histology revealed synovial hyperplasia, inflammatory cell infiltration, and cartilage damage. Micro-CT confirmed significant bone erosion, evidenced by an increased bone surface/bone volume ratio (P<0.001). Serum levels of IL-1β, TNF-α, and IL-6 were significantly elevated. Allopurinol treatment effectively lowered uric acid and alleviated joint swelling and bone erosion.

**Conclusion:**

This study successfully established and systematically characterized the phenotypic features of a CGA rat model that integrates chronic hyperuricemia, acute uric acid fluctuations, and local MSU crystal deposition. Phenotypically, this model recapitulates the core features of human CGA, including joint swelling, bone erosion, and functional impairment, thereby providing an experimental platform for investigating chronic joint damage induced by multiple interacting factors.

## Introduction

Gout is a crystal-induced arthritis caused by the deposition of monosodium urate (MSU) crystals, which is closely associated with purine metabolism disorders leading to reduced uric acid excretion and/or increased production (1, 2). In some patients without regular treatment, recurrent acute attacks progress into a chronic arthritic phase, known as chronic gouty arthritis (CGA), characterized by elevated serum uric acid levels, tophi formation, bone erosion, joint deformity, stiffness, and limited mobility (3, 4). These structural changes profoundly affect patients’ quality of life; however, research into the pathological mechanisms and therapeutic strategies for CGA is largely limited by the lack of an ideal animal model that accurately recapitulates the disease progression.

CGA is a multi-system disorder involving metabolic and immunological pathways. Elevated serum uric acid represents the fundamental etiology of CGA (5). MSU crystals constitute the core pathological driver of both the initiation and progression of CGA, directly promoting disease chronicity and structural damage through multiple mechanisms (6, 7). Furthermore, fluctuations in uric acid levels serve as a significant trigger for acute gout flares: rapid increases may lead to sudden MSU crystal deposition within joints, provoking inflammatory responses (8), while abrupt decreases can cause surface dissolution of pre-formed crystals, releasing pro-inflammatory substances and inducing attacks (9). However, currently established animal models are largely limited to those for hyperuricemia (10, 11) and acute gouty arthritis (12), which cannot be applied to studies of systemic uric acid metabolism or gout-related bone pathology.

Given the importance of a reliable CGA animal model for studying disease mechanisms and testing new drugs, we established a rat model of chronic gout. Hyperuricemia was induced by a diet containing potassium oxonate and yeast, while recurrent intra-articular MSU crystal deposition was achieved by intermittent hypoxanthine administration followed by repeated MSU injections into the ankle joint and paw. At the phenotypic level, this model recapitulates some of the etiological and clinical features of chronic gouty arthritis, thereby providing a preliminary basis for exploratory research in mechanistic studies and comparative evaluations.

## Materials and methods

### MSU crystal preparation

Accurately weigh 400 mg of uric acid and place it into 80 mL of distilled water. Dissolve thoroughly using a magnetic stirrer, with the temperature maintained at 60–80 °C to accelerate dissolution. Slowly add 1 mol/L NaOH solution dropwise while monitoring the pH with a precision pH meter, adjusting the pH to 8.9. Allow the solution to crystallize at room temperature for 24 hours. Collect the crystals on filter paper and wash three times with anhydrous ethanol. Accelerate drying using a microwave oven at high power. Transfer the MSU crystals to a centrifuge tube and sterilize by dry heat at 180 °C for 2 hours. Prior to use, grind the MSU crystals and pass them through a 40-μm cell strainer, suspending in sterile physiological saline to prepare an MSU crystal suspension (25 mg/mL).

### Animal model preparation and grouping

The ethical approval for this study was obtained from the Ethics Committee of Beijing University of Chinese Medicine (Approval No. 2024101705-4068).

**Figure 1:**
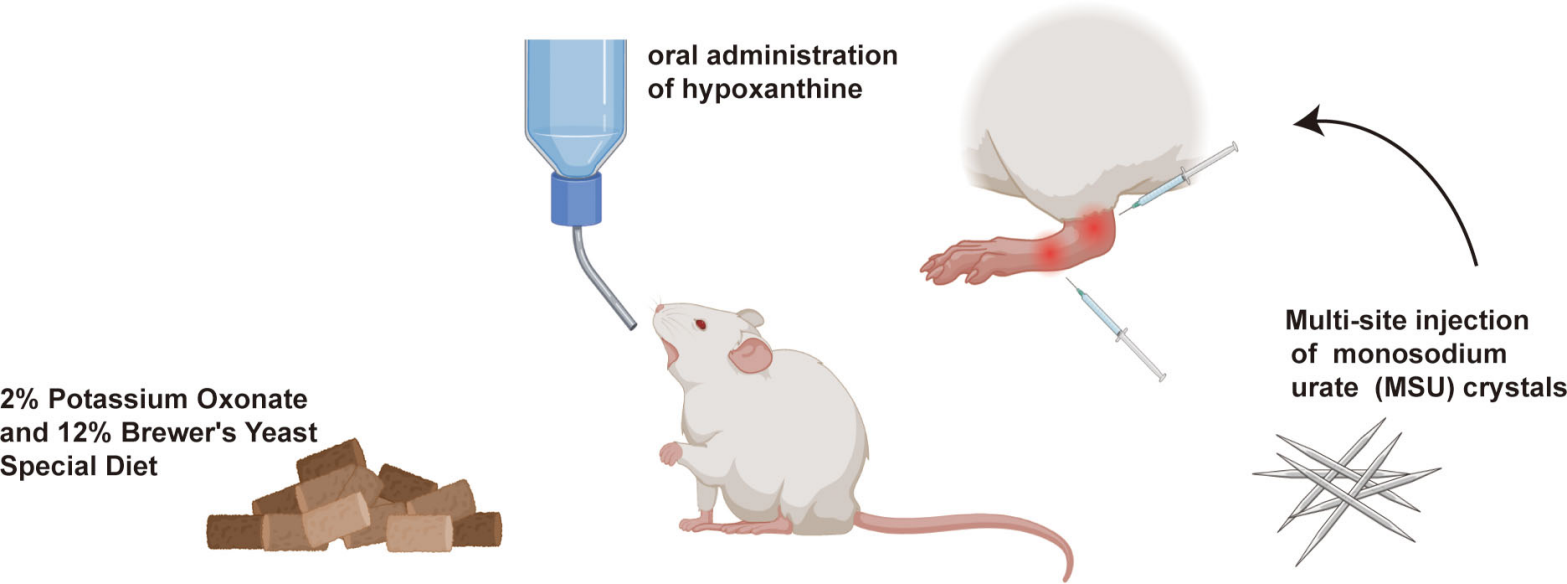
Schematic diagram of the study protocol.

A total of 36 healthy, SPF-grade, 4-week-old male SD rats (purchased from Sibeifu (Beijing) Biotechnology Co., Ltd.) were housed under standard conditions at a constant temperature of 21 ± 1 °C with a 12-hour light/dark cycle. After one week of acclimatization, a total of 36 rats were randomly divided into two groups using a random number table: a blank control group (n = 12) and an experimental group (n = 24). The blank control group was fed a standard diet, while the experimental group received a special diet containing 2% potassium oxonate and 12% yeast (formulated by Beijing BioPike Biotechnology Co., Ltd.) to induce hyperuricemia. After 2 weeks, the rats were again randomly divided into a model group (n = 12) and an allopurinol treatment group (n = 12) using a random number table. The model group received intra-articular injections of 25 mg/mL MSU crystals (100 μL each into the right ankle joint cavity and the center of the footpad) twice weekly to repeatedly induce gouty arthritis. Concurrently, 2 hours before each ankle joint injection, the model group was orally administered a hypoxanthine solution (100 mg/kg) to simulate fluctuations in serum uric acid levels, thereby establishing a chronic hyperuricemia combined with gouty arthritis model (**Figure 1**). The allopurinol treatment group additionally received daily oral administration of allopurinol tablets at 9.3 mg/kg/day. To ensure consistency, the model group received oral administration of an equivalent volume of saline. The blank control group received daily oral administration of an equivalent volume of saline and unilateral intra-articular injections of saline (100 μL each into the ankle joint cavity and footpad center) for 10 weeks, to simulate the effects of the gavage and injection procedures. Sample collection and related analyses were performed one week after the final joint cavity injection.

### Blinding procedures

All data collection and analysis were performed by researchers who were blinded to the group assignments. Specifically, joint swelling measurements, inflammation scoring, and behavioral tests were conducted by two independent investigators unaware of the group allocation. Sample labeling was managed by another researcher, and data compilation and analysis were carried out by a fourth investigator, ensuring blinding throughout the entire process.

### Serum uric acid measurement

To dynamically monitor changes in serum uric acid levels, blood samples were collected from the retro-orbital venous plexus at weeks 2, 4, 8, and 10 of the special diet feeding regimen. Sampling was performed immediately before and 1 hour after each hypoxanthine gavage administration. At the end of the experimental period, terminal blood was collected via the abdominal aorta. All blood samples were centrifuged to obtain serum. Serum uric acid concentrations were measured using an automated biochemical analyzer at the Clinical Laboratory of Dongzhimen Hospital.

### Assessment of joint swelling

The degree of ankle swelling was assessed by measuring the ankle diameter. Prior to intra-articular injection of the MSU suspension, the ankle joint was circumferentially marked with a permanent marker. On the day of sampling, the diameter was measured at the marked site using a digital micrometer caliper. To minimize measurement variability, each measurement was repeated three times, and the average value was calculated.

### Joint inflammation scoring

On the day of sampling, evaluations were performed. The degree of joint inflammation was assessed according to the following criteria:0 (Normal): No edema or swelling; bony landmarks visible; normal skin color.1 (Mild): Mild redness and swelling confined to the ankle joint; skin reddish, slightly increased local temperature; bony landmarks still visible.2 (Moderate): Pronounced redness and swelling confined to the ankle joint; locally increased temperature; bony landmarks obscured.3 (Severe): Severe swelling and erythema involving the entire paw; inability to flex the ankle; bony landmarks completely obscured.

### Hindlimb hanging test

To evaluate joint pain, functional impairment, and muscle weakness associated with chronic gouty arthritis, a hindlimb hanging test was conducted. Each rat was placed on a cage lid, allowing all four paws to grip the grid. The lid was then inverted, suspending the rat in a head-down position. The latency until the first instance where either hindlimb lost its grip and released from the grid was recorded. The testing order for all animals was randomized, and the procedure was performed by an experimenter blinded to the group assignments. Each rat underwent two familiarization trials to acclimatize to the test conditions, followed by three formal trials conducted at 20-minute intervals. The mean hanging time from the three formal trials was calculated for analysis.

### Histopathological examination

Following abdominal aorta blood collection, the hind paws and ankle regions were dissected and fixed in 4% paraformaldehyde solution. The specimens were then decalcified in 14% EDTA decalcifying solution, with the degree of decalcification monitored every two days until the bones became pliable. After embedding, tissues were sectioned at a thickness of 4–5 μm using a microtome. Tissue sections were stained with hematoxylin and eosin (H&E) to evaluate the severity of inflammation, synovial hyperplasia, bone erosion, and cartilage damage. Cartilage damage was further assessed by Safranin O-Fast Green staining.

### Imaging analysis

The fixed hind limbs of rats were scanned using micro-computed tomography (Micro-CT) to evaluate the ankle joints of each group. The region of interest (ROI) was delineated by a single investigator (blinded to group assignment) using blinded analysis. The ROI encompassed the area from the distal edge of the tibial epiphyseal plate to the proximal talus, covering the entire ankle joint region. Segmentation thresholds for bone and soft tissue were determined using a global thresholding method (automatically calculated by CTan software using Otsu’s algorithm, with independent adjustments for each sample and subsequent averaging). The extracted bone parameters included bone volume (BV, mm^3^), bone surface area (BS, mm^2^), and bone surface area/bone volume ratio (BS/BV, mm^-1^). Each sample was measured three times, and the mean value was calculated. To ensure reproducibility, ROI delineation and threshold settings were repeated by the same operator after a one-week interval, and the intraclass correlation coefficient (ICC) between the two measurements was >0.9.

### Measurement of inflammatory cytokines

Serum levels of IL-1β, IL-6, and TNF-α were quantified in rats from each group using commercially available ELISA kits, following the manufacturer’s recommended dilution protocols and assay procedures.

### Statistical analysis

Data were analyzed using GraphPad Prism version 10. All quantitative data are expressed as the mean ± standard deviation (SD). One-way analysis of variance (ANOVA) was used to compare joint swelling, hanging test latency, serum levels of IL-1β, TNF-α, and IL-6, the BS/BV ratio, and histopathological scores. The Kruskal-Wallis test was employed for analyzing inflammatory scores. Statistical significance is denoted as *p < 0.05, **p < 0.01, ***p < 0.001, and ****p < 0.0001.

## Results

### Typical manifestations of CGA in model rats

Rats in the normal group exhibited good mental status, glossy fur, normal food intake, significant weight gain, and normal urination and defecation. Compared with the normal group, at 6 weeks, rats in the model group and allopurinol group showed swelling of the ankle joints and plantar region, with the affected foot contacting the ground but the ankle slightly flexed. Their walking speed was slower than that of normal rats, appetite was slightly reduced, body weight increased, and urination and defecation remained normal. At 12 weeks, rats displayed dull fur, swelling of the ankle and plantar areas, palpable hard nodules locally, lifting of the affected foot off the ground, three-legged lameness, significantly reduced walking speed, and normal urination and defecation. No mortality occurred in any of the three groups during the rearing period.

### Assessment of joint swelling, inflammation, and behavioral function

**Figure 2:**
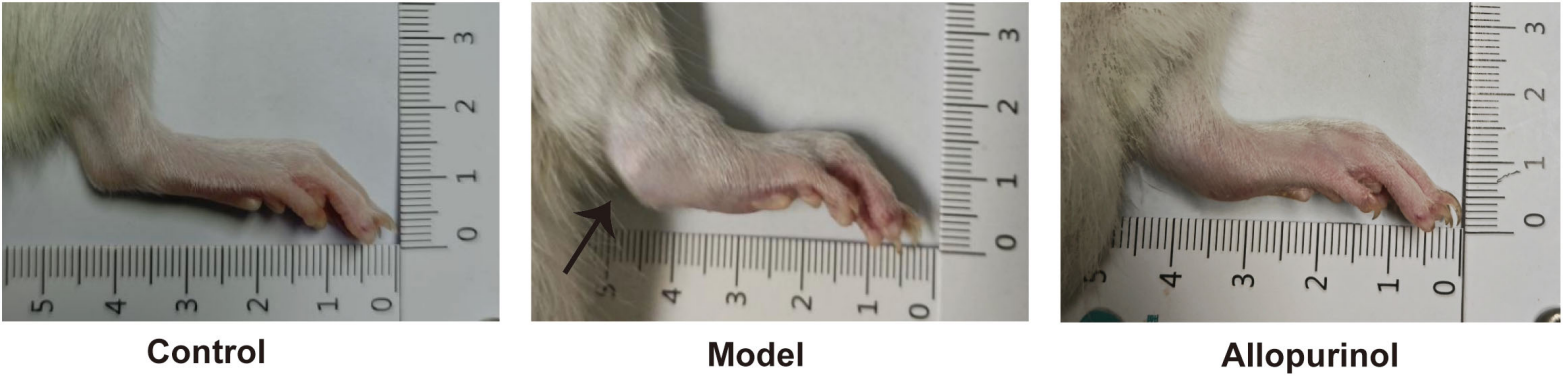
Gross appearance of rat ankle joints. Black arrows indicate tophus formation.

Induced by a special diet, intermittent oral administration of hypoxanthine, and intraplantar/infrared injections of MSU, the model group exhibited macroscopic features of chronic gouty arthritis, including swelling and deformation of the ankle joints and plantar regions. Severe cases in the model group showed the formation of whitish subcutaneous tophi (**Figure 2**). In the allopurinol-treated group, swelling of the ankle and plantar areas was observed, but no tophi were detected. The blank control group displayed no significant swelling or other inflammatory responses.

**Figure 3:**
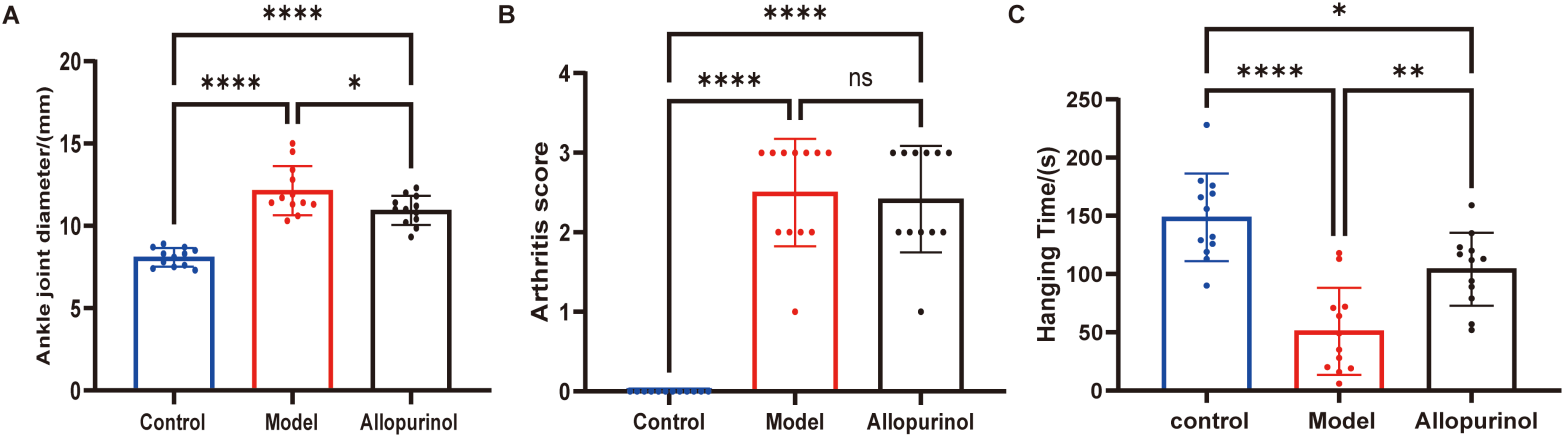
Functional and inflammatory assessments in the chronic gouty arthritis model. **(A)** Joint swelling was quantified by ankle diameter (mm). **(B)** Arthritis severity was evaluated using a clinical inflammation score. **(C)** Functional impairment was assessed via the hanging test (seconds). Data are represented as the mean ± SD, with n=12 animals per group. *p<0.05, **p<0.01, ****p<0.0001.

Compared with the blank control group, the model group demonstrated a significantly increased degree of joint swelling (P < 0.0001). Although swelling in the allopurinol-treated group remained greater than that in the blank control group (P < 0.0001), it was significantly reduced compared to the model group (P < 0.05) (**Figure 3A**). No statistically significant difference was observed in joint inflammation scores between the allopurinol-treated and model groups (P > 0.05) (**Figure 3B**). In the behavioral test, the model group showed a significantly shortened suspension time compared to the blank control group (P < 0.0001). In contrast, the allopurinol-treated group exhibited a significantly prolonged suspension time relative to the model group (P < 0.01), indicating that allopurinol treatment effectively ameliorated joint dysfunction (**Figure 3C**).

### Sustained hyperuricemia and acute uric acid fluctuations

**Figure 4:**
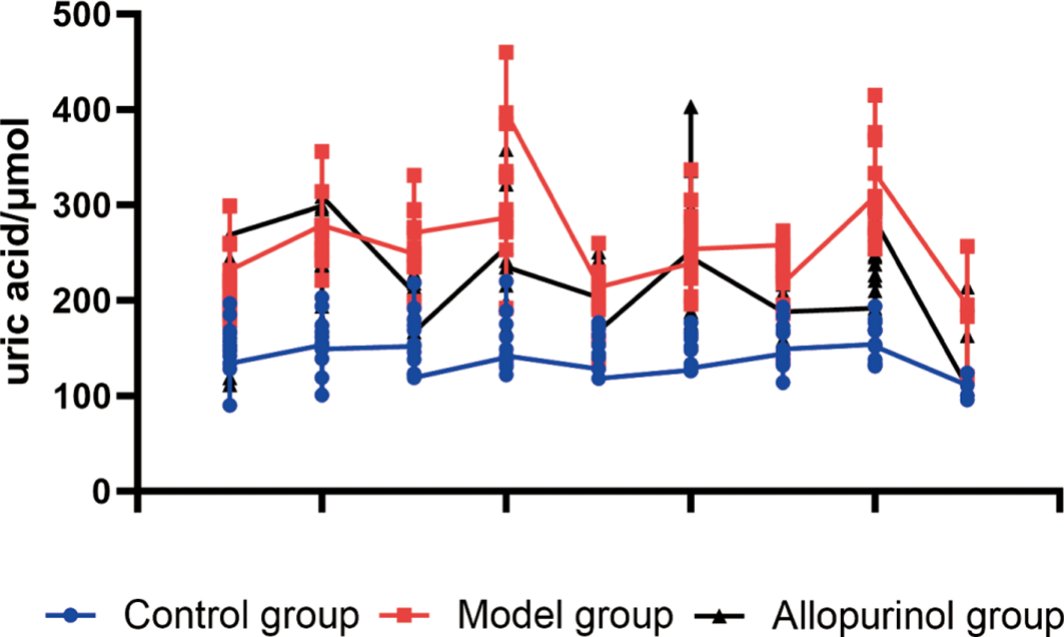
Temporal profile of serum uric acid levels. This figure shows the changes in serum uric acid at weeks 2, 4, 8, and 10 (before and after hypoxanthine gavage) and at the end of the study (week 12), capturing the acute urate fluctuations and chronic elevation induced by the regimen.

Following feeding with a special diet containing 2% potassium oxonate and 12% yeast, the baseline serum uric acid levels in both the model group and the allopurinol-treated group were significantly higher than those in the blank control group. After the oral administration of hypoxanthine, both groups exhibited an acute peak in uric acid fluctuation. However, under continuous allopurinol intervention, the serum uric acid levels at all time points—including both the peak fluctuation and steady-state levels—were significantly lower than those in the model group at corresponding times (P < 0.01), demonstrating elevated serum uric acid in the CGA rats and the efficacy of allopurinol in effectively reducing uric acid levels (**Figure 4**).

### Histopathological evidence of bone damage in model rats

**Figure 5:**
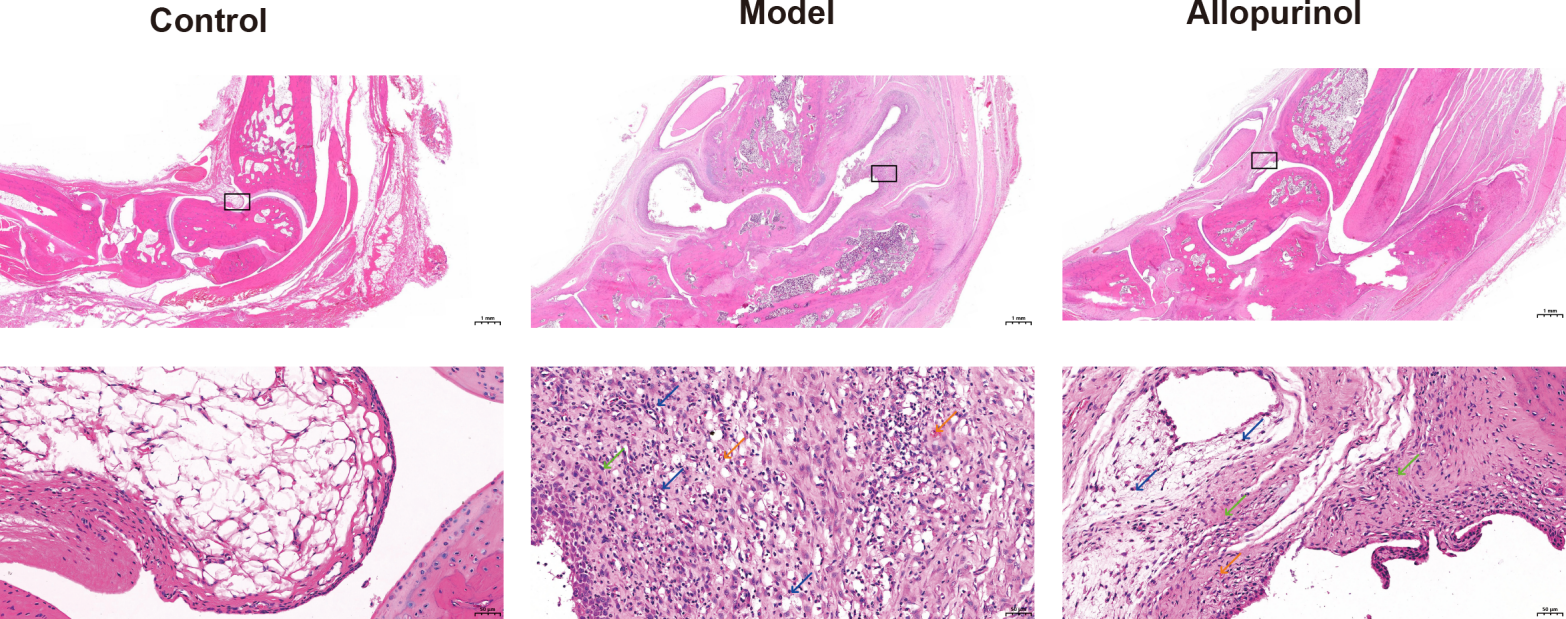
Histopathological analysis of ankle joints (H&E staining). Control: Ordered synovial lining cells, loose connective tissue.Model: Moderate synovial hyperplasia (green arrows), mixed inflammatory infiltration (blue arrows), neovascularization (orange arrows).Allopurinol: Mild synovial hyperplasia (green arrows), minimal inflammation (blue arrows), limited neovessels (orange arrows).Black boxes indicate magnified areas.

H&E staining revealed that in the blank control group, the synovial lining cells were arranged in an orderly manner, and the subsynovial connective tissue exhibited a loose structure. The articular cartilage surface was smooth, with chondrocytes uniformly arranged and no obvious hyperplasia, bone erosion, or inflammatory cell infiltration. In contrast, the model group showed marked synovial hyperplasia, disorganized collagen fibers, and extensive infiltration of granulocytes, lymphocytes, and macrophages, accompanied by neovascularization. Bone tissue was eroded, and the cartilage structure was disrupted and replaced by proliferative fibrous connective tissue. Allopurinol treatment alleviated the above inflammatory responses and bone damage (**Figure 5**).

**Figure 6:**
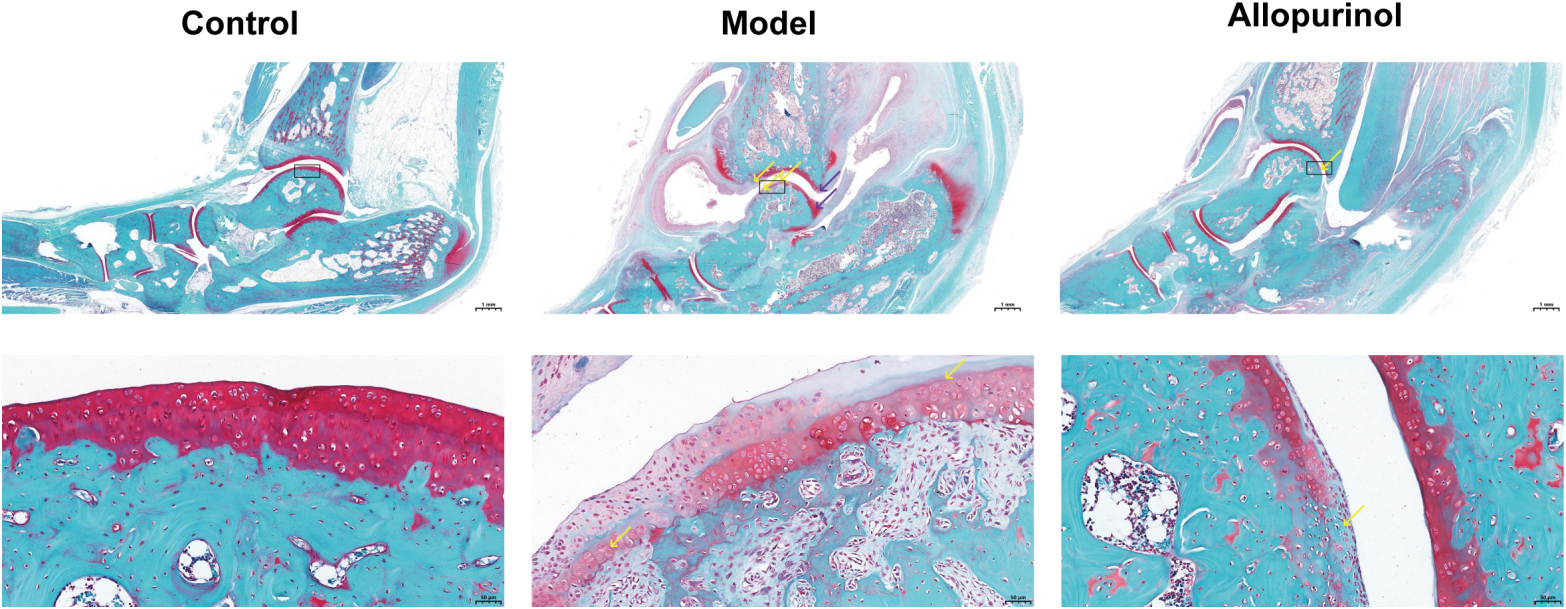
Histopathological analysis of ankle joints (Safranin O-Fast Green staining). Control: The articular cartilage surface is smooth with well-organized chondrocyte arrangement and intact proteoglycan staining, indicating normal cartilage structure. Model: Multiple areas of the tibial and talar cartilage show erosion by fibrous connective tissue (yellow arrows), accompanied by reduced chondrocyte density and diminished proteoglycan staining (loss of red color). Focal chondrocyte hyperplasia is observed in the tibia and talus (purple arrows). Allopurinol: Focal loss of proteoglycan staining is noted in the medial talar cartilage (yellow arrow), indicating localized cartilage damage. The black boxes indicate the magnified areas.

Safranin O–Fast Green staining indicated that the blank control group displayed smooth articular cartilage surfaces, regularly arranged chondrocytes, and normal morphology. In the model group, multiple cartilage areas in the ankle joint were eroded by fibrous connective tissue, accompanied by a reduction in chondrocyte number, loss of matrix staining, and chondrocyte hyperplasia on the joint surface. Allopurinol treatment ameliorated cartilage structural destruction and matrix loss compared with the model group (**Figure 6**).

### Quantitative micro-CT analysis of bone erosion

**Figure 7:**
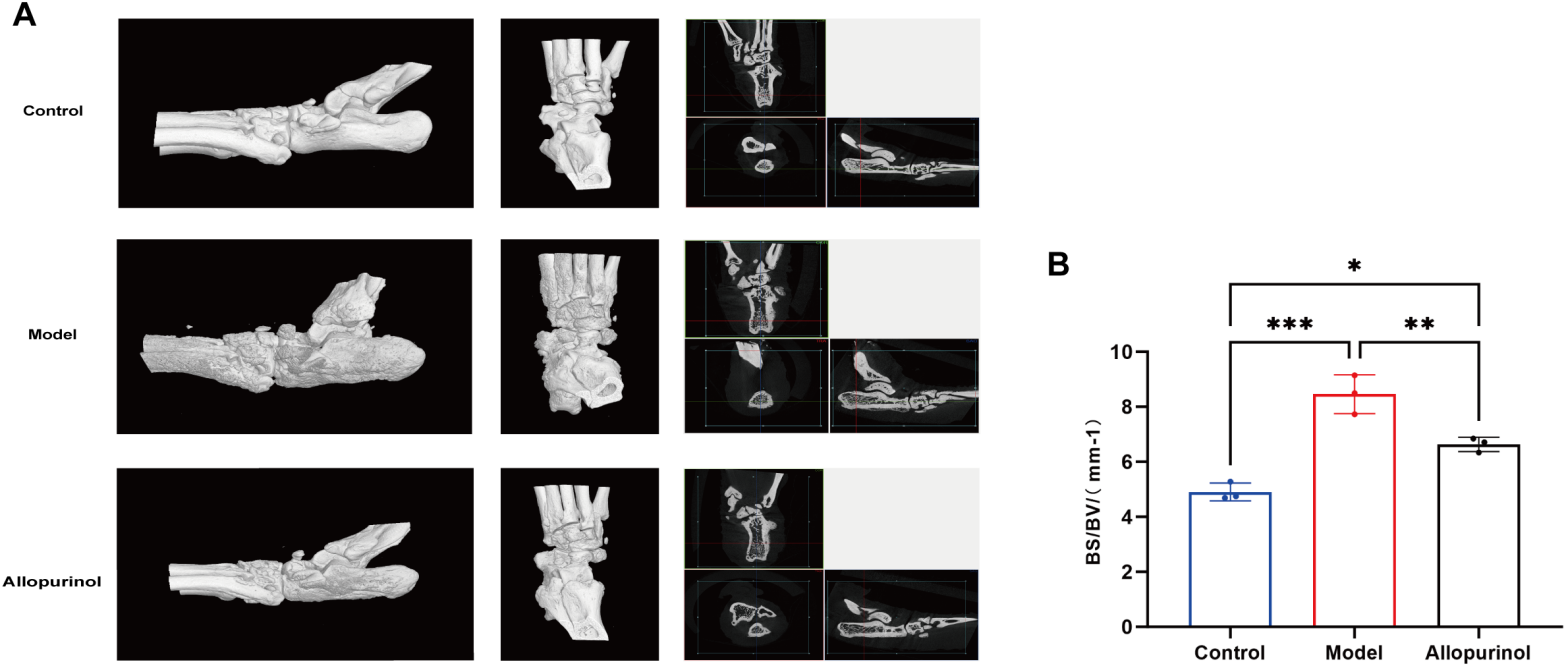
Representative micro-CT evaluation of ankle joints. **(A)** Three-dimensional reconstruction images. The control group displays intact bone structure with smooth articular surfaces. The model group exhibits pronounced bone erosion, joint space narrowing, and increased surface roughness. Allopurinol treatment attenuated these pathological changes, indicating reduced bone erosion and improved joint architecture. **(B)** Quantitative analysis of bone surface area/bone volume (BS/BV) ratio. Data are represented as the mean ± SD, with n=3 animals per group. *p<0.05, ***p < 0.001 were calculated using one-way ANOVA.

To assess whether the model exhibits typical bone erosion characteristics of chronic gouty arthritis, the severity of bone erosion in rat hind paw and ankle tissues was evaluated by micro-CT imaging. Quantitative micro-CT analysis revealed that the bone surface area/bone volume (BS/BV) ratio was significantly higher in the model group than in the blank control group (P < 0.01), indicating impaired trabecular structure and severe bone erosion. Allopurinol treatment significantly reduced the BS/BV ratio compared to the model group (P < 0.05), although it did not fully restore the ratio to the level of the blank control group (P < 0.05). Three-dimensional reconstructed images visually demonstrated that allopurinol treatment markedly ameliorated bone surface roughness and joint space destruction compared to the model group (**Figure 7**).

### Elevated serum levels of inflammatory cytokines

**Figure 8:**
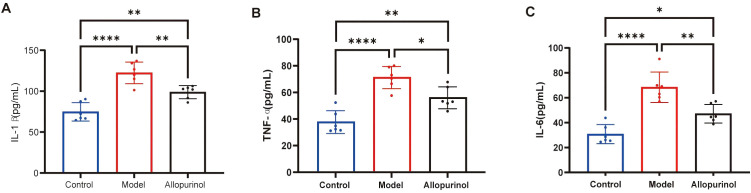
Serum levels of pro-inflammatory cytokines. Compared with the blank control group, the model group exhibited significantly elevated serum levels of **(A)** IL-1β, **(B)** TNF-α, and **(C)** IL-6. Allopurinol treatment reduced the concentrations of these pro-inflammatory cytokines. Data are represented as the mean ± SD, with n=6 animals per group. *p<0.05, **p<0.01, ****p<0.0001 were calculated using one-way ANOVA.

Compared with the blank control group, the model group exhibited significantly elevated levels of IL-1β, TNF-α, and IL-6. Allopurinol treatment reduced the concentrations of these proinflammatory cytokines, suggesting that urate-lowering therapy may help alleviate the systemic inflammatory state driven by MSU crystals (**Figure 8**).

## Discussion

The rationale for model construction in this study is as follows: A stable hyperuricemic background was established using a combination of potassium oxonate and a yeast-supplemented diet. Potassium oxonate, possessing a purine-like structure, competitively inhibits uricase activity in rodents and represents a classic approach for modeling hyperuricemia in these species (13, 14). Yeast, as a natural source of purines, mimics increased uric acid production induced by a high-purine diet in humans and also enhances xanthine oxidase (XOD) activity, thereby accelerating uric acid generation (15, 16). MSU deposition triggers inflammatory responses (17–19), inhibits chondrocyte autophagy, impedes tissue repair, and directly damages joints by causing irreversible cartilage injury and loss of joint function (20), ultimately driving the transition from intermittent acute flares to chronic arthritis. The direct application of MSU crystals in our model ensures sustained inflammatory stimulation and tissue destruction. Furthermore, multi-site injections into the plantar and ankle regions induce bone erosion and subcutaneous nodule formation over a wider area, thereby enhancing the success rate of model establishment. Clinical observations have confirmed that rapid fluctuations in uric acid levels serve as an important trigger for recurrent gout attacks. In this animal model, intra-articular MSU crystal injection was performed following oral administration of hypoxanthine, which induced acute fluctuations in uric acid levels. This approach mimics the endogenous or exogenous triggers of acute flares in gout patients.

In this study, we successfully established a rat model integrating chronic metabolic abnormalities, acute uric acid fluctuations, and local crystal deposition by combining a special diet containing potassium oxonate and yeast, intermittent hypoxanthine gavage, and repeated intra-articular and plantar injections of MSU crystals. The model exhibited significant joint swelling, elevated inflammatory scores, and reduced hanging time, indicating chronic inflammation, pain, and functional impairment. Histopathological examination further revealed inflammatory nodules, chondrocyte proliferation, cartilage fissures, synovial hyperplasia, and inflammatory cell infiltration in the ankle joints. Micro-CT imaging revealed typical bone erosion and joint destruction, indicating a key pathological transition that distinguishes this model from acute gout models. At the molecular level, serum levels of pro-inflammatory cytokines, including IL-1β, TNF-α, and IL-6, were significantly elevated in the model group, suggesting a persistent chronic inflammatory response associated with MSU crystal stimulation. Furthermore, we treated the established model with allopurinol, a first-line clinical agent. Allopurinol intervention significantly reduced serum uric acid levels, accompanied by attenuated joint swelling, improved bone erosion, and decreased inflammatory cytokine levels, indicating that this model exhibits the expected response to first-line clinical urate-lowering therapy.

In recent years, research on animal models of gout has remained a persistent challenge in the field. Current modeling approaches predominantly rely on the injection of monosodium urate (MSU) crystals into joint cavities or subcutaneous air pouches to induce acute gouty arthritis (21, 22). However, these models fail to recapitulate key clinical manifestations of chronic gouty arthritis (CGA), including bone erosion, tophus formation, and sustained hyperuricemia (23, 24). One study successfully simulated spontaneous tophus formation using a combination of a high-fat diet, intraplantar acetic acid injection, and intraperitoneal administration of potassium oxonate (25). Nevertheless, the deposition rate of MSU crystals in that model was only 37.9%, representing a relatively low success rate. Moreover, the absence of longitudinal comparisons with the natural disease course of human gout limits its applicability for studying the dynamic progression of the disease. With advances in genetic engineering, several knockout models of hyperuricemia have been developed, including URAT1 (26), ABCG2 (27) and GLUT9 (28) deficiency models. Although these animal models exhibit significantly elevated serum uric acid levels, they are often associated with premature mortality due to severe nephropathy, rendering them unsuitable for long-term studies of chronic gouty arthritis requiring extended observation periods. In contrast, the present model successfully integrates chronic metabolic dysregulation with acute crystal-induced inflammation, combining the metabolic background characteristic of hyperuricemia (HUA) models with the acute inflammatory features of acute gouty arthritis (AGA) models. Through repeated multi-site injections, this model effectively recapitulates the transition from acute flares to the chronic arthritis phase, simulating advanced CGA manifestations such as tophus formation and bone erosion. Consequently, this model may serve as a basis for exploratory investigations into the molecular and immunological mechanisms underlying the progression from intermittent acute attacks to a chronic persistent state in CGA. Furthermore, it may serve as a potential tool for evaluating novel therapeutic strategies aimed at preventing bone erosion, inhibiting tophus formation, or modulating uric acid fluctuations.

Certainly, this study has several limitations. First, the absence of a significant difference in joint inflammation scores between the allopurinol-treated and model groups may be attributed to the visual scoring system’s focus on acute inflammatory signs. While allopurinol treatment indicated the model’s expected response to urate-lowering therapy, its therapeutic mechanism requires further investigation. Second, the Micro-CT analysis of bone destruction was based on a small sample size (n=3 per group); future studies should include larger samples for validation. Third, this study examined only the combined effects of hyperuricemia, uric acid fluctuations, and repeated MSU injection, without isolating individual factors or including direct comparisons with established models (e.g., repeated MSU injection alone or hyperuricemia alone). Therefore, the current evidence does not allow us to draw conclusions regarding the relative performance or translational advantage of the proposed model. Finally, as MSU crystals were exogenously injected rather than endogenously formed, this model is not suitable for studying the initial process of crystal formation.

In summary, this study represents a proof-of-concept investigation demonstrating the feasibility of a multi-factor integrated modeling strategy for CGA. We have successfully developed and characterized a novel rat model of chronic gouty arthritis that integrates hyperuricemia, uric acid fluctuations, and local MSU crystal deposition. This model recapitulates the clinical manifestations and pathological features of human CGA at the phenotypic level and may serve as a useful tool for further research. Our subsequent research will focus on utilizing this model to further elucidate the pathogenesis of CGA and to screen for therapeutic agents capable of effectively halting its chronic progression.

## Data Availability

The original contributions presented in the study are included in the article/supplementary material. Further inquiries can be directed to the corresponding author.
